# De-escalation implementation in low-risk papillary thyroid cancer: a nationwide survey

**DOI:** 10.1530/EC-25-0796

**Published:** 2026-05-15

**Authors:** Grigoris Effraimidis, Eleni Sazakli, Olga Karapanou, Katerina Saltiki, Marina A Michalaki

**Affiliations:** ^1^ Faculty of Medicine, School of Health Sciences, University of Thessaly, Larissa, Greece; ^2^ Department of Endocrinology and Metabolic Diseases, Larissa University Hospital, Larissa, Greece; ^3^ Faculty of Medicine, School of Health Science, University of Patras, Patras, Greece; ^4^ Endocrine Department, NIMTS Veteran’s Hospital, Athens, Greece; ^5^ Endocrine Unit, Department of Clinical Therapeutics, National and Kapodistrian University, Athens, Greece

**Keywords:** papillary thyroid carcinoma, low risk, low-to-intermediate risk, web-survey, de-escalation

## Abstract

**Background:**

Over the past decade, the management of papillary thyroid carcinoma (PTC) has become increasingly individualized, with less aggressive approaches recommended for low-risk diseases. However, the real-world implementation of these recommendations remains limited.

**Objective:**

This study aims to assess real-world management patterns and therapeutic preferences regarding surgical extent, radioactive iodine (RAI) use, and thyrotropin (TSH) levels in low- and low-to-intermediate-risk PTC through a nationwide survey.

**Methods:**

A nationwide web-based survey was conducted among members of the Greek Endocrine Society (25% response rate) between November 2023 and April 2024. The questionnaire comprised demographic items, 12 clinical scenarios, and a final section exploring general reasons for nonadherence to clinical guidelines; this report focuses on eight scenarios related to low- and low-to-intermediate-risk papillary thyroid carcinoma.

**Results:**

For an 18 mm intrathyroidal low-risk PTC, 67.7% of respondents recommended total thyroidectomy, while 51.8% favored adjuvant RAI. When reclassified as low-to-intermediate risk, 92.5% endorsed RAI, often at higher doses (70 mCi), particularly among more experienced and those practicing in large cities. TSH suppression targets in case of excellent response varied: nearly half selected 0.5–2.0 μU/mL for low-risk PTC, but most favored tighter suppression (0.1–0.5 μU/mL) in low-to-intermediate-risk scenarios. Senior endocrinologists prefer traditional approaches. Barriers to guideline adherence included limited access to molecular and ultrasonography testing, a shortage of experienced surgeons outside major centers, and skepticism regarding guideline safety.

**Conclusion:**

Our findings underscore persistent practice variation driven by professional experience and local healthcare infrastructure, underscoring the need for targeted local implementation strategies, particularly outside major urban centers.

## Introduction

Over the past decades, the management of papillary thyroid carcinoma (PTC) has shifted from a standard approach to a more tailored strategy. In particular, international guidelines (1, 2, 3) for low- and low-to-intermediate-risk PTC emphasize the role of de-escalation. Despite strong recommendations from professional societies, such as the American Thyroid Association (ATA) (1) and the European Thyroid Association (ETA) (2), the adoption of de-escalation remains heterogeneous worldwide, influenced by factors such as physician specialty, clinical experience, healthcare infrastructure, and cultural attitudes toward cancer treatment. In our country, real-world data on how endocrinologists incorporate de-escalation into their management of PTC remain scarce.

In this context, we conducted a nationwide survey among endocrinologists, focusing on their therapeutic preferences for low- and low-to-intermediate-risk PTC, their approach to active surveillance for low-risk microPTC (4), and their management strategies for thyroid nodules with atypia of undetermined significance (AUS) cytology (5). This study aims to explore endocrinologists’ decision-making processes regarding surgical extent, radioactive iodine (RAI) administration, and TSH suppression targets in low- and low-to-intermediate-risk PTC. Decisions were assessed using standardized clinical scenarios.

## Materials and methods

### Study design and recruitment

This is the third part of a web-survey conducted among members of the Greek Endocrine Society examining the management of thyroid nodules with AUS cytology, low-risk microPTC, and low- and low-to-intermediate-risk PTCs. Herein, we present the nationwide preferences of endocrinologists regarding the management of low- and low-to-intermediate-risk PTCs.

As previously reported, a total of 809 endocrinologists (376 men and 433 women) were invited to participate, aiming to obtain a sample size representative of at least 20% of our society members (4, 5). Briefly, our endocrine society distributed the survey to its members, providing a study overview and a link to the online questionnaire, which was hosted on the Survey Legend platform. Participation was voluntary, with confidentiality and anonymity ensured. Recruitment occurred between November 2023 and April 2024, with a reminder sent in January 2024. A pilot test–retest study was conducted with 15 endocrinologists, who completed the questionnaire twice over a 5-month interval. These pilot responses were used solely to evaluate questionnaire reliability and were excluded from the final study analysis.

The questionnaire consisted of three sections. The first section collected demographic data, assessed self-reported confidence in managing patients with thyroid nodules or PTC on a 5-point Likert scale, and identified preferred educational tools for their management. The second section of the survey presented 12 clinical scenarios, which were grouped into the three thematic categories: two scenarios addressed second-line management of thyroid nodules with AUS (5); two focused on the management of low-risk microPTC (4); and eight dealt with the management of low- and low-to-intermediate-risk PTC. Each scenario required endocrinologists to choose one of three or four management approaches. The third section included a final question addressing the main reasons for nonadherence to guidelines across all 12 clinical scenarios.

In this paper, we present the third group of clinical scenarios, involving a 60-year-old woman with papillary carcinoma (Supplemental Material).

### Ethics approval and consent to participate

Before completing the survey, all participants were informed about the purpose of the study, assured that participation was voluntary, and that confidentiality and anonymity would be maintained. By proceeding to complete the survey, participants provided their informed consent for their responses to be analyzed and published. All procedures were conducted in accordance with the ethical standards of the institutional and national research committees and with the Declaration of Helsinki (2013 revision), and all authors confirmed that appropriate processes have been followed.

### Data analysis

Descriptive statistics summarized the demographic data of the respondents. Group proportions were calculated for categorical variables. Differences in proportions were tested using the chi-square test. The nonparametric Kolmogorov–Smirnov test compared the geographical distributions of the entire population and the participants’ sample. Questionnaire reliability was assessed using Cohen’s kappa coefficient, a measure of agreement, based on endocrinologists’ pilot-phase responses at two time points. Spearman’s rho correlation coefficient was calculated to assess bivariate associations. Group comparisons of numerical variables were performed by the nonparametric Mann–Whitney *U* test. Multivariate binary logistic regression models (selected answer vs all other options) evaluated the effect of the participants’ characteristics: sex, years since residency (ordinal variable), location of clinical practice (large cities vs other), employment sector (public vs private), self-confidence level (ordinal variable), and annual number of PTC patients (nominal variable, split at 75th percentile). Models were constructed with the forward stepwise (likelihood ratio) method; factors with *P* < 0.150 were retained. Statistical significance was set at *α* = 0.05. Analyses were performed with IBM SPSS v.28 (IBM Corp., USA).

## Results

### Study participants and their characteristics

A total of 201 endocrinologists (response rate 25%) participated in the study. Sex and geographic distribution of respondents were representative of the overall endocrine society membership (Kolmogorov–Smirnov *Z* test, *P* = 1.000). Most of the participants were women (58.0%), having 11–30 years of post-residency experience (56.7%), living in the two largest cities (68.2%), and working in the private sector (76.6%). The annual average number of PTC patients monitored by the participants ranged from 1 to 180, with the 75th percentile at 15. In terms of self-confidence in managing patients with thyroid nodules and PTC most respondents (61.5%) declared to be very confident. (Supplement 1 (see section on Supplementary materials given at the end of the article)). The questionnaire demonstrated good test–retest reliability in the pilot sample (Cohen’s kappa = 0.804, *P* < 0.001).

Absolute confidence was reported more frequently by men (19.0%) than by women (6.0%) (*P* = 0.003), while years of experience were associated with higher self-confidence levels (*r* = 0.338, *P* < 0.001), but only among women. No significant difference in self-confidence was observed between physicians working in the public versus private sectors (*P* = 0.096). Regarding the most effective educational tool for managing thyroid nodules or PTC, 33.8% of respondents identified a combination of conferences, clinical practice, and literature as the most helpful, while 26.3% favored clinical practice alone (as previously reported). These preferences showed no significant differences based on sex (*P* = 0.174), employment sector (*P* = 0.308), years of residency (*P* = 0.058), or location of clinical practice (*P* = 0.729).

### Responses to the clinical scenarios

The distribution of responses provided for the eight scenarios is presented in Table 1. Specific variables were examined for their association with each response in each scenario. These included sex, years since residency (ordinal variable), location of clinical practice (large cities vs other), employment sector (public vs private), self-confidence level (ordinal variable), and annual number of PTC patients (nominal variable, dichotomized at the 75th percentile). Variables retained in the final models are presented in Table 2.

**Table 1:** Distribution of responses in the clinical scenarios.

Clinical scenarios	Available options	Responses (%)
1. A 60-year-old woman has papillary carcinoma 18 mm, intraparenchymal and without suspicious cervical RL or other nodules on cervical ultrasound (low-risk). What would you recommend?	Lobectomy	9.9
	Total thyroidectomy	67.7
	Total thyroidectomy and prophylactic central lymph node dissection	22.4
2. To the woman of scenario 1, would you administer postoperative RAIs?	Very likely	27.4
	Likely	24.4
	Less likely	32.3
	Unlikely	15.9
3. To the woman of scenario 1, if you decided to administer RAI postoperatively, what would be the dose?	30 mCi	54.2
	50 mCi	26.9
	70 mCi	10.4
	100 mCi	8.5
4. After 1 year the baseline levels of Tg in the woman of scenario 1 are <0.2 ng/mL (with negative anti-Tg) and cervical ultrasound without findings (excellent response). What is the goal of TSH?	<0.1 μU/mL	8.9
	0.1–0.5 μU/mL	44.8
	0.5–2.0 μU/mL	46.3
5. A 60-year-old woman has undergone total thyroidectomy for classic 18 mm papillary carcinoma and has 3 microscopically infiltrated central compartment LNs (1–2 mm) without vascular infiltration or microscopic extrathyroid expansion (low-to-intermediate risk). Would you administer postoperative RAIs?	Very likely	71.1
	Likely	21.4
	Less likely	7.0
	Unlikely	0.5
6. A 60-year-old woman has undergone total thyroidectomy for classic papillary carcinoma of 18 mm and has no known infiltrated LN or no vascular infiltration while she has microscopic extrathyroid expansion (low-to-intermediate risk). Would you administer postoperative RAIs?	Very likely	67.6
	Likely	24.9
	Less likely	6.0
	Unlikely	1.5
7. To the woman of scenarios 5 and 6, if you decided to administer RAI postoperatively, what would be the dose?	30 mCi	13.9
	50 mCi	26.9
	70 mCi	30.8
	100 mCi	28.4
8. After 1 year the baseline levels of Tg in the woman of scenario 5 are <0.2 ng/mL (with negative anti-Tg) and cervical ultrasound without findings (excellent response). What is the goal of TSH?	<0.1 μU/mL	26.9
	0.1–0.5 μU/mL	53.2
	0.5–2.0 μU/mL	19.9

**Table 2:** Effect of study population characteristics on selection of responses.

Scenario 1					
No association of any response with any characteristics. Models not significant					
Scenario 2					
***A) Very likely/likely***	Very likely/likely	Less likely/unlikely	**OR** *	**95% CI** ^†^	***P-value***
Years since residency	*n*	*n*			
1–5	*10*	*25*	1.0		
6–10	*19*	*14*	**3.62**	**1.29–10.15**	***0.015***
11–30	*66*	*48*	**3.69**	**1.57–8.66**	***0.003***
>30	*9*	*9*	**2.67**	**0.80–8.86**	***0.109***
Scenario 3					
No association of any response with any characteristics. Models not significant					
Scenario 4					
***A) <0.1 μU/mL***					
No association with any characteristics. Model not significant					
***B) 0.1–0.5 μU/mL***	0.1–0.5 μU/mL	Other responses	**OR**	**95% CI**	***P-value***
Years since residency	*n*	*n*			
1–5	*7*	*28*	1.0		
6–10	*16*	*17*	**4.24**	**1.39–12.95**	***0.011***
11–30	*55*	*59*	**4.04**	**1.55–10.55**	***0.004***
>30	*12*	*6*	**9.00**	**2.40–33.70**	***0.001***
***C) 0.5–2.0 μU/mL***	0.5–2.0 μU/mL	Other responses	**OR**	**95% CI**	***P-value***
Years since residency	*n*	*n*			
1–5	*27*	*8*	1.0		
6–10	*15*	*18*	**0.22**	**0.08–0.66**	***0.007***
11–30	*47*	*67*	**0.20**	**0.08–0.49**	***<0.001***
>30	*3*	*15*	**0.05**	**0.01–0.24**	***<0.001***
Scenario 5					
***A) Very likely***	Very likely	Other responses	**OR**	**95% CI**	***P-value***
Years since residency	*n*	*n*			
1–5	*19*	*16*	1.0		
6–10	*29*	*4*	**6.04**	**1.73–21.09**	***0.005***
11–30	*81*	*33*	**2.08**	**0.94–4.63**	***0.072***
>30	*14*	*4*	**2.92**	**0.79–10.76**	***0.108***
***B) Likely***	Likely	Other responses	**OR**	**95% CI**	***P-value***
Years since residency	*n*	*n*			
1–5	*13*	*22*	1.0		
6–10	*2*	*31*	**0.11**	**0.02–0.56**	***0.007***
11–30	*24*	*90*	**0.45**	**0.19–1.05**	***0.066***
>30	*4*	*14*	0.50	0.13–1.87	*0.303*
***C) Less likely***					
No association with any characteristics. Model not significant					
***D) Unlikely***					
No association with any characteristics. Model not significant					
Scenario 6					
No association of any response with any characteristics. Models not significant					
Scenario 7					
***A) 30 mCi***					
No association with any characteristics. Model not significant					
***B) 50 mCi***					
No association with any characteristics. Model not significant					
***C) 70 mCi***	70 mCi	Other responses	**OR**	**95% CI**	***P-value***
Location of clinical practice	*n*	*n*			
Other areas	*12*	*50*	1.0		
Largest cities	*49*	*86*	**2.35**	**1.14–4.86**	***0.021***
N of patients with PTC annually					
<15	*41*	*109*	1.0		
≥15	*21*	*28*	**1.81**	**0.91–3.60**	***0.091***
***D) 100 mCi***	100 mCi	Other responses	**OR**	**95% CI**	***P-value***
N of patients with PTC annually	*n*	*n*			
<15	*48*	*102*	1		
≥15	*8*	*41*	**0.44**	**0.19–1.02**	***0.056***
Scenario 8					
***A) <0.1 μU/mL***	<0.1 μU/mL	Other responses	**OR**	**95% CI**	***P-value***
Years since residency	*n*	*n*			
1–5	4	31	1.0		
6–10	11	22	**6.06**	**1.47–24.98**	***0.013***
11–30	32	82	**4.11**	**1.16–14.64**	***0.029***
>30	7	11	**6.48**	**1.39–30.13**	***0.017***
Location of clinical practice					
Other areas	*11*	*51*	1.0		
Largest cities	*42*	*93*	**2.21**	**1.02–4.79**	***0.046***
N of patients with PTC annually					
<15	44	106	1.0		
≥15	9	40	**0.48**	**0.21–1.10**	***0.082***
***B) 0.1–0.5 μU/mL***	0.1–0.5 μU/mL	Other responses	**OR**	**95% CI**	***P-value***
Location of clinical practice	*n*	*n*			
Other areas	40	22	1.0		
Largest cities	66	69	**0.53**	**0.29–0.99**	***0.047***
***C) 0.5–2.0 μU/mL***					
No association with any characteristics. Model not significant					

* OR, odds ratio.

^†^ CI, confidence interval. Italicized column presents p-values. Significant associations are depicted with bold. Variables included in the models: sex (male, female), years of post-residency experience (1–5, 6–10, 11–30, >30), location of clinical practice (large cities vs other areas), employment sector (public vs private sector), self-confidence level (moderately confident, very confident, absolutely confident), and the annual number of patients with PTC (<15 patients vs ≥15 patients).

In a 60-year-old woman with an 18 mm intraparenchymal PTC classified as low-risk, most endocrinologists (67.7%) would recommend total thyroidectomy (scenario 1) and consider postoperative RAI administration as very likely or likely (51.8%; scenario 2), preferably at a dose of 30 mCi (54.2%; scenario 3). Demographic and other participant characteristics were not associated with any of the responses in scenario 1 (Table 2). In scenario 2, response options were grouped as follows: ‘very likely’ and ‘likely’ were combined into a single ‘likely’ category, while ‘less likely’ and ‘unlikely’ were merged into ‘unlikely’. In the subsequent analysis, only years of specialty were associated with the preference for postoperative RAI administration. Specifically, endocrinologists with more than 5 years of post-residency experience were nearly 3–4 times more likely to recommend RAI administration, compared to those with fewer than 5 years of experience (Table 2). However, no association was found between physicians' characteristics and the dose of RAI to be administered (scenario 3). Regarding the target TSH level in the same woman with low-risk PTC and excellent response to initial therapy (scenario 4), responses were almost evenly split between 0.1–0.5 μU/mL (44.8%) and 0.5–2.0 μU/mL (46.3%). Endocrinologists with more than 5 years post-residency opted for a target TSH of 0.1–0.5 μU/mL (Table 2).

When the above PTC was reclassified as low-to-intermediate risk, due to either microscopic lymph node infiltration (scenario 5) or minimal extrathyroidal extension (scenario 6), 92.5% of physicians indicated they would likely or very likely administer RAI. Specifically, in scenario 5, the majority (71.1%) strongly favored postoperative RAI, and this preference was significantly associated with the number of years since completing residency. In contrast, in scenario 6, the models did not identify any association between the number of years since completing residency or any other participant characteristics and the selected response. Regarding the RAI dose (scenario 7), responses were nearly evenly distributed. Endocrinologists practicing in the two largest cities were 2.4 times more likely to select the 70 mCi dose, compared to those practicing outside major urban centers, while those managing 15 or more patients with PTC annually also had higher odds to administer 70 mCi (Table 2).

Finally, in scenario 8, which described a woman with low-to-intermediate-risk PTC and an excellent response to treatment, 53.2% of respondents reported targeting a TSH level of 0.1–0.5 μU/mL, while 26.9% would aim for a TSH level below 0.1 μU/mL. More experienced endocrinologists were more likely to select the lower TSH target (<0.1 μU/mL) compared to those with 1–5 years since completing residency. Practice location also influenced responses: endocrinologists practicing in large cities were 2.2 times more likely to choose this lower TSH target. In contrast, those managing a higher volume of PTC patients were less likely to aim for a TSH level below 0.1 μU/mL.

### Reasons for noncompliance

A total of 94 endocrinologists (46.8% of the total 201 respondents) expressed doubts about the guidelines issued by scientific societies, raising concerns regarding patient safety. Among the 201 respondents, the most frequently cited barriers to compliance were the inability to perform reliable ultrasound (71 respondents, 35.3%) and molecular testing (69 respondents, 34.3%). Other reported challenges included insufficient information (22.4%) and a lack of experienced surgeons (17.9%). Notably, endocrinologists practicing outside of the metropolitan area of the country were significantly more likely to cite the shortage of experienced surgeons as a reason for noncompliance compared to those working within the capital (*P* = 0.009).

## Discussion

This nationwide web-survey underscores the persistence gap between international guideline recommendations and real-world management of low- and low-to-intermediate-risk PTC. While international trends emphasize de-escalation to reduce overtreatment, our findings show that most endocrinologists still prefer conventional management, namely total thyroidectomy and routine RAI administration, irrespective of individual risk. Our data uniquely emphasizes the role of healthcare infrastructure, geographic disparities, and cultural attitudes in shaping clinical decision-making.

The preference for total thyroidectomy (68%) for an 18 mm intrathyroidal low-risk PTC (scenario 1) reflects longstanding practice patterns. Although the 2015 ATA guidelines endorsed lobectomy as an adequate option for selected low-risk PTC (6), our nationwide data indicate that de-escalation remains limited in routine practice. Nevertheless, the use of thyroid lobectomy for low-risk DTC remains heterogeneous across regions. Among 420 Turkish multidisciplinary responders, total thyroidectomy was the predominant approach (65%) even for classical unilateral microcarcinoma (7). Similarly, a Spanish web-based survey of 135 endocrinologists and 143 surgeons revealed low preference for hemithyroidectomy in low-risk PTC (<50% even for 1.1–2.5 cm tumors) (8). A survey conducted among 320 surgeons from the United States revealed that a large proportion of them do not support lobectomy for patients with low-risk PTC measuring 1–4 cm (9). On the other hand, the extensive experience with de-escalated surgical procedures in countries such as Japan and Korea (10, 11, 12) aligns with the preferences of physicians in these regions. A Korean study surveyed surgeons and endocrinologists on surgical preferences for 1.5–2.5 cm PTC and showed that both groups favored hemithyroidectomy for tumors without extrathyroidal extension (preference for hemithyroidectomy: 66–96% of endocrinologists and 74–96% of surgeons). However, endocrinologists were less likely than surgeons to choose hemithyroidectomy when minimal or gross anterior ETE was present (13). Our findings showed that demographic and professional characteristics were not associated with the choice between lobectomy and total thyroidectomy in this scenario. In other studies, the choice between total thyroidectomy and lobectomy among endocrinologists and surgeons is influenced by several factors, such as tumor size (9, 14), the experience of surgeons (9), and institutional strategy or national context (15). In McDow *et al.*’s survey of US general surgeons and otolaryngologists, both tumor size and surgeon experience significantly influenced surgical preferences. The proportion recommending lobectomy declined with increasing tumor size, from 43.1% for a 1.1 cm PTC to 11% for a 3 cm tumor. High-volume surgeons more often endorsed lobectomy. In contrast, in the Spanish survey, being a high-volume specialist was associated with a lower preference for hemithyroidectomy (8) and an analysis of 3,199 thyroid cancer patients between 2013 and 2020 revealed that the 2015 ATA guidelines significantly increased lobectomy rates among low-volume surgeons, but not among high-volume surgeons (16).

Postoperative RAI therapy in low- and low-to-intermediate-risk patients with PTC tumors >1 cm remains a controversial issue, particularly from the perspective of nuclear medicine (17). In our survey, more than half of the respondents would recommend RAI for an 18-mm low-risk PTC (scenario 1), increasing to 92.5% when classified as low-to-intermediate risk (scenarios 5 and 6). Preference for RAI was more common among endocrinologists with greater post-residency experience, while those practicing in major urban centers and managing higher PTC volumes tended to favor higher doses (70 mCi). According to the recent ETA Consensus Statement, RAI treatment is not indicated for PTC tumors <1 cm (whether unifocal or multifocal) (2). For larger tumors, postoperative neck ultrasound and serum thyroglobulin (Tg) levels should be taken into consideration when deciding on RAI therapy. In these scenarios, however, both postoperative neck ultrasound and serum Tg levels were missing. In contrast, among those selecting to administer RAI, a preference for low doses (30–50 mCi) was dominant (81.1%), suggesting significant guideline adherence regarding dosage de-escalation (scenario 3). This trend for standard management may reflect persistent concerns about recurrence and medicolegal concerns at a time when evidence from randomized trials on RAI omission was limited. Notably, only the 3-year results of the ESTIMABL2 trial were available, demonstrating the non-inferiority of omitting postoperative RAI in selected low-risk patients (18). Since then, longer-term and complementary evidence has become available. The 5-year follow-up of ESTIMABL2 (19) included patients with pT1 tumors, N0 or Nx nodal status, who underwent total thyroidectomy with or without prophylactic central lymph node dissection, and had no postoperative suspicious findings on neck ultrasonography. This analysis confirmed the sustained non-inferiority of RAI omission, with no excess of structural or biological events, reinforcing the safety of this approach in appropriately selected low-risk patients. In parallel, another RCT, the IoN trial (20) provided additional evidence in a wider group of patients, including patients with pT2 and pT3a tumors and central nodal involvement. With a median follow-up of 6.6–6.8 years, the 5-year recurrence-free rate, defined by structural events, was similar between the two groups. Although ESTIMABL2 has faced criticism, particularly regarding the definition of functional event, which may lead to misinterpretation and potential bias in favor of the non-RAI group (21), the combined findings of these two independent RCTs provide robust and complementary evidence. Together, they support the safe omission of routine RAI ablation after total thyroidectomy in selected DTC patients with pT1–pT2 tumors, N0 nodal status, and no other adverse features.

Thus, while many clinicians still recommend RAI for patients with low-risk or low-to-intermediate-risk DTC, others in Europe, the United States, and Australia do not (22, 23, 24). This discrepancy might be driven by multiple factors, including physician specialty, personal perspectives on acceptable recurrence risk, volume of thyroid cancer cases treated, and institutional protocols (25).

When considering TSH suppression targets after an optimal treatment response in low-risk PTC, endocrinologists exhibited greater adherence to guidelines. Nearly half of the endocrinologists selected a target TSH range of 0.5–2.0 μU/mL for a low-risk PTC patient with an excellent response to therapy (scenario 4), aligning with current guideline recommendations. In the scenario involving a patient with low-to-intermediate-risk differentiated thyroid carcinoma and an excellent response (scenario 8), 53% of respondents selected a TSH goal of 0.1–0.5 μU/mL. As in the previous scenarios, endocrinologists with more years of experience tended to prefer standard therapeutic approaches. These results are in line with a survey from the United States conducted among endocrinologists and surgeons where a significant proportion (80%) were likely to recommend TSH suppression for patients with intermediate-risk PTC, while this likelihood decreased but was still remarkable for low-risk (48%) and very low-risk (30%) PTC patients (26). Notably, the study did not specify whether the clinical scenarios described referred to patients who had achieved an excellent response to initial therapy. The study also revealed heterogeneity across specialties and experience levels, as surgeons and high-volume clinicians were less likely to recommend suppression, while physicians perceiving higher recurrence risk tended to apply it even in very low-risk cases.

While this study provides valuable insights into endocrinologists’ attitudes toward de-escalation in low-to-intermediate-risk PTC, certain limitations must be acknowledged. Notably, 58.2% of survey respondents were over 50 years old, and greater years of experience – closely correlated with older age – were associated with a preference for more conventional approaches. This aligns with our observation in the first part of our survey, in which more senior endocrinologists were more likely to diverge from recently adopted practices for nodules with AUS cytology (5). As previously noted (5), this pattern may partly reflect ‘clinical inertia’; however, it may also indicate greater risk awareness or cumulative exposure to more complex cases, leading to a more cautious management approach. Moreover, as with most surveys, respondent fatigue, particularly toward the final questions, cannot be ruled out, although the average questionnaire completion time was approximately 10 min. Finally, as part of a broader project, the final question on noncompliance with guidelines captured only general attitudes across all clinical scenarios and was not designed to identify barriers specific to surgical extent, RAI administration, or TSH suppression decisions; therefore, these findings should be interpreted with caution. Future research should include more targeted questions to better assess practice settings, resource availability, and barriers to implementing de-escalation strategies.

It should be noted that, in the absence of national clinical practice guidelines for DTC in our country, clinical decision-making relies primarily on those of the ATA and ETA. Despite their wide acceptance, the implementation of these guidelines may be shaped by local healthcare infrastructure, resource availability, and cultural attitudes toward cancer treatment.

To date, only a few surveys have explored the real-world implementation of de-escalation strategies recommended by the ATA and ETA over the past decade in the management of low-risk PTC. Although our survey was conducted before the release of the 2025 ATA guidelines (3), the management of the clinical scenarios we presented herein did not differ between the 2015 and 2025 recommendations. Furthermore, the ‘low-to-intermediate risk’ category addressed in our study corresponds to the newly introduced classification in the updated 2025 ATA risk stratification system. Our findings emphasize the importance of developing implementation of local strategies that accompany guideline dissemination (27).

## Supplementary materials



## Declaration of interest

The authors declare that there is no conflict of interest that could be perceived as prejudicing the impartiality of the work reported.

## Funding

This research received no external funding. The publication fees of this manuscript were financed by the Research Council of the University of Patras.

## Author contribution statement

GE: conceptualization (supporting), review and editing (equal); ES: methodology (lead), formal analysis (lead); OK: conceptualization (supporting); KS: conceptualization (equal); MM: conceptualization (lead), review and editing (equal), project administration, supervision (lead). All authors read and approved the final manuscript.

## Ethics approval and consent to participate

This study was reviewed and approved by the Ethics Committee of the University of Patras (Approval No. 15770/28-07-2023).

## References

[1] Haugen BR, Alexander EK, Bible KC, et al. 2015 American Thyroid Association Management Guidelines for adult patients with thyroid nodules and differentiated thyroid cancer: the American Thyroid Association Guidelines task force on thyroid nodules and differentiated thyroid cancer. Thyroid 2016 26 1–133. (10.1089/thy.2015.0020) 26462967 PMC4739132

[2] Pacini F, Fuhrer D, Elisei R, et al. 2022 ETA Consensus Statement: what are the indications for post-surgical radioiodine therapy in differentiated thyroid cancer? Eur Thyroid J 2022 11 e210046. (10.1530/ETJ-21-0046) 34981741 PMC9142814

[3] Ringel MD, Sosa JA, Baloch Z, et al. 2025 American Thyroid Association Management Guidelines for adult patients with differentiated thyroid cancer. Thyroid 2025 35 841–985. (10.1177/10507256251363120) 40844370 PMC13090833

[4] Effraimidis G, Sazakli E, Karapanou O, et al. Active surveillance for low-risk papillary thyroid microcarcinoma: a web-survey on clinician readiness for change. Eur Thyroid J 2025 14 e250013. (10.1530/ETJ-25-0013) 40019776 PMC11949526

[5] Sazakli E, Karapanou O, Sykiotis GP, et al. Nationwide web survey on implementing the 2023 ETA guidelines for second-line management of thyroid nodules with atypia of undetermined significance. Hormones 2025 24 1003–1011. (10.1007/s42000-025-00698-4) 40721567 PMC12678575

[6] Matsuura D, Yuan A, Harris V, et al. Surgical management of low-/intermediate-risk node-negative thyroid cancer: a single-institution study using propensity matching analysis to compare thyroid lobectomy and total thyroidectomy. Thyroid 2022 32 28–36. (10.1089/thy.2021.0356) 34861772 PMC8792497

[7] Makay Ö, Özdemir M, Giles Senyurek Y, et al. Surgical approaches for papillary microcarcinomas: Turkey’s perspective. Turkish J Surg 2018 34 89–93. (10.5152/turkjsurg.2018.3596) PMC6048650 30023969

[8] Díez JJ, Parente P & Durán-Poveda M. Surgical management of low-risk papillary thyroid cancer in real life in Spain: a nationwide survey of endocrine neck surgeons and endocrinologists. Endocrine 2023 83 422–431. (10.1007/s12020-023-03488-3) 37592163

[9] McDow AD, Saucke MC, Marka NA, et al. Thyroid lobectomy for low-risk papillary thyroid cancer: a national survey of low- and high-volume surgeons. Ann Surg Oncol 2021 28 3568–3575. (10.1245/s10434-021-09898-9) 33939048 PMC11975426

[10] Lee J, Park JH, Lee CR, et al. Long-term outcomes of total thyroidectomy versus thyroid lobectomy for papillary thyroid microcarcinoma: comparative analysis after propensity score matching. Thyroid 2013 23 1408–1415. (10.1089/thy.2012.0463) 23509895

[11] Matsuzu K, Sugino K, Masudo K, et al. Thyroid lobectomy for papillary thyroid cancer: long-term follow-up study of 1,088 cases. World J Surg 2014 38 68–79. (10.1007/s00268-013-2224-1) 24081532

[12] Jeon YW, Gwak HG, Lim ST, et al. Long-term prognosis of unilateral and multifocal papillary thyroid microcarcinoma after unilateral lobectomy versus total thyroidectomy. Ann Surg Oncol 2019 26 2952–2958. (10.1245/s10434-019-07482-w) 31264119

[13] Cheon YI, Shin SC, Lee M, et al. Survey of Korean head and neck surgeons and endocrinologists for the surgical extent of 1.5 and 2.5 cm papillary thyroid carcinoma. Gland Surg 2022 11 1744–1753. (10.21037/gs-22-326) 36518800 PMC9742055

[14] Barbaro D, Basili G & Materazzi G. Total thyroidectomy vs lobectomy in differentiated thyroid cancer: is there a reasonable size cut-off for decision? A narrative review. Gland Surg 2021 10 2275–2283. (10.21037/gs-21-242) 34422598 PMC8340330

[15] Kovatch KJ, Hoban CW & Shuman AG. Thyroid cancer surgery guidelines in an era of de-escalation. Eur J Surg Oncol 2018 44 297–306. (10.1016/j.ejso.2017.03.005) 28385370 PMC5600641

[16] Ellsworth BL, Sinco B, Matusko N, et al. Examining national guideline changes associated with hemithyroidectomy rates by surgeon volume. J Surg Res 2023 283 858–866. (10.1016/j.jss.2022.11.037) 36915013

[17] Petranović Ovčariček P, Kreissl MC, Campenni A, et al. SNMMI/EANM practice guideline vs ETA consensus statement: differences and similarities in approaching differentiated thyroid cancer management—the EANM perspective. Eur J Nucl Med Mol Imag 2022 49 3959–3963. (10.1007/s00259-022-05935-1) 35947178

[18] Leboulleux S, Bournaud C, Chougnet CN, et al. Thyroidectomy without radioiodine in patients with low-risk thyroid cancer. N Engl J Med 2022 386 923–932. (10.1056/NEJMoa2111953) 35263518

[19] Leboulleux S, Bournaud C, Chougnet CN, et al. Thyroidectomy without radioiodine in patients with low-risk thyroid cancer: 5 years of follow-up of the prospective randomised ESTIMABL2 trial. Lancet Diabetes Endocrinol 2025 13 38–46. (10.1016/S2213-8587(24)00276-6) 39586309

[20] Mallick U, Newbold K, Beasley M, et al. Thyroidectomy with or without postoperative radioiodine for patients with low-risk differentiated thyroid cancer in the UK (IoN): a randomised, multicentre, non-inferiority trial. Lancet 2025 406 52–62. (10.1016/S0140-6736(25)00629-4) 40543520

[21] Tuncel M, Ovčariček PP & Giovanella L. Five-year follow-up of ESTIMABL2 trial: a critical appraisal. Eur J Nucl Med Mol Imag 2025 52 4753–4756. (10.1007/s00259-025-07375-z) 40439713

[22] Wijewardene A, Gild M, Nylén C, et al. Change in practice of radioactive iodine administration in differentiated thyroid cancer: a single-centre experience. Eur Thyroid J 2021 10 408–415. (10.1159/000516358) 34540711 PMC8406251

[23] Park KW, Wu JX, Du L, et al. Decreasing use of radioactive iodine for low-risk thyroid cancer in California, 1999 to 2015. J Clin Endocrinol Metab 2018 103 1095–1101. (10.1210/jc.2017-02269) 29267880

[24] Jacobs D, Breen CT, Pucar D, et al. Changes in population-level and institutional-level prescribing habits of radioiodine therapy for papillary thyroid cancer. Thyroid 2021 31 272–279. (10.1089/thy.2020.0237) 32811347

[25] McDow AD, Roman BR, Saucke MC, et al. Factors associated with physicians’ recommendations for managing low-risk papillary thyroid cancer. Am J Surg 2021 222 111–118. (10.1016/j.amjsurg.2020.11.021) 33248684 PMC8262457

[26] Papaleontiou M, Chen DW, Banerjee M, et al. Thyrotropin suppression for papillary thyroid cancer: a physician survey study. Thyroid 2021 31 1383–1390. (10.1089/thy.2021.0033) 33779292 PMC8558057

[27] Barth JH, Misra S, Aakre KM, et al. Why are clinical practice guidelines not followed? Clin Chem Lab Med 2016 54 1133–1139. (10.1515/cclm-2015-0871) 26650076

