# Supplementary material for: De-escalation implementation in low-risk papillary thyroid cancer: a nationwide survey
Source: Endocr Connect. 2026 May 15;15(5):e250796. doi: 10.1530/EC-25-0796 (PMC13187569; doi:10.1530/EC-25-0796)
Supplement: Supplementary file 1 [file supplementary_materials.pdf]

Supplementary Table 1: The Distributed Questionnaire and Reported Preference Rates Among Greek Endocrinologists

Section 1. Epidemiological data

1. Sex
a. Male
b. female
2. Age (years)
a. 30-39
b. 40-49
c. 50-59
d. 60-69
e. >70
3. Years since obtaining an endocrinology specialty
a. 1-5
b. 6-10
c. 11-30
d. >30
4. What is your workplace?
a. Private sector
b. Public sector
5. In which Prefecture do you practice specialty?.....................
6. How many patients with DRF do you monitor on average per year?...........................
7. How confident do you feel about the management of patients with thyroid nodule or papillary thyroid carcinoma?
a. Not at all confident
b. A little bit confident
c. Moderately confident
d. Very confident
e. Absolutely confident
8. Which of the following do you think would help you manage patients with thyroid nodule or papillary thyroid carcinoma?
a. Conferences -
b. Clinical tutorials - case studies in tertiary hospitals
c. Bibliography

Section 2. Clinical scenarios

Scenario 1. A 60-year-old woman has papillary carcinoma 18 mm, intraparenchymal and without suspicious cervical RL or other nodules on cervical ultrasound (low-risk). What would you recommend?
a. Lobectomy
b. Total thyroidectomy
c. Total thyroidectomy and prophylactic central lymph node dissection

Scenario 2. In a 60-year-old woman who has undergone total thyroidectomy for classical papillary carcinoma of 18 mm and has no known infiltrated cervical MN, vascular infiltration or extrathyroid expansion (low-risk). Would you administer postoperative RAIs?
a. it is very likely
b. likely
c. less likely
D. unlikely
Scenario 3. To the woman in scenario 1, if you decided to administer RAI postoperatively, what would be the dose?
a. 30 mCi
b. 50 mCi
c. 70 mCi
d. 100 mCi
Scenario 4. A 60-year-old woman has undergone total thyroidectomy for classic 18mm papillary carcinoma and has no known infiltrated cervical RL, vascular infiltration or extrathyroid expansion (low-risk). After one year the baseline levels of Tg are <0.2ng/mL (with negative anti-Tg) and cervical ultrasound without findings (excellent response). What is the goal of TSH:
a. TSH < 0.1μU/mL
b. TSH: 0.1 - 0.5μU/mL
c. TSH: 0.5-2.0 μU/mL
Scenario 5. A 60-year-old woman has undergone total thyroidectomy for classic 18mm papillary carcinoma and has 3 microscopically infiltrated central compartment LNs (1-2mm) without vascular infiltration or microscopic extrathyroid expansion (low to intermediate risk). Would you administer postoperative RAIs?
a. it is very likely
b. likely
c. less likely
D. Unlikely
Scenario 6. A 60-year-old woman has undergone total thyroidectomy for classic papillary carcinoma of 18 mm and has no known infiltrated LN or no vascular infiltration while she has microscopic extrathyroid expansion (low to intermediate risk). Would you administer postoperative RAIs?
a. it is very likely
b. likely
c. less likely
d. Unlikely
Scenario 7. To the women of scenario 5, if you decided to administer RAI postoperatively, what would be the dose?
a. 30mCi
b. 50mCi
c. 70mCi
d. 100mCi
Scenario 8. A 60-year-old woman has undergone total thyroidectomy for classic 18mm papillary carcinoma and has 3 microscopically infiltrated central compartment RLs (1-

2mm) without vascular infiltration or extrathyroid expansion (low to intermediate risk). After one year the baseline levels of Tg are <0.2ng/mL (with negative anti-Tg) and cervical ultrasound without findings (excellent response). What is the goal of TSH:

a. TSH<0.1μU/mL

b. TSH:0.1-0,5μU/mL

c. TSH:0.5-2.0 μU/mL

Section 3. Reasons for non-compliance

9. **What are the main reasons(s) for non-compliance with the Guidelines?**

a. Incomplete information

b. I am not convinced by the instructions and I am concerned about my patient's safety

c. Inability to perform reliable cervical ultrasonography

d. Inability to perform molecular control

e. lack of experienced surgeons throughout the country
